# The plausible reason why the length of 5' untranslated region is unrelated to organismal complexity

**DOI:** 10.1186/1756-0500-4-312

**Published:** 2011-08-27

**Authors:** Chun-Hsi Chen, Hsuan-Yu Lin, Chia-Lin Pan, Feng-Chi Chen

**Affiliations:** 1 Division of Biostatistics and Bioinformatics, Institute of Population Health Sciences, National Health Research Institutes, Zhunan, Miaoli County, 350 Taiwan; 2 Department of Life Science, National Chiao-Tung University, Hsinchu, 300 Taiwan; 3 Department of Dentistry, Chinese Medical University, Taichung, 404 Taiwan

## Abstract

**Background:**

Organismal complexity is suggested to increase with the complexity of transcriptional and translational regulations. Supporting this notion is a recent study that demonstrated a higher level of tissue-specific gene expression in human than in mouse. However, whether this correlation can be extended beyond mammals remains unclear. In addition, 5' untranslated regions (5'UTRs), which have undergone stochastic elongation during evolution and potentially included an increased number of regulatory elements, may have played an important role in the emergence of organismal complexity. Although the lack of correlation between 5'UTR length and organismal complexity has been proposed, the underlying mechanisms remain unexplored.

**Results:**

In this study, we select the number of cell types as the measurement of organismal complexity and examine the correlation between (1) organismal complexity and transcriptional regulatory complexity; and (2) organismal complexity and 5'UTR length by comparing the 5'UTRs and multiple-tissue expression profiles of human (*Homo sapiens*), mouse (*Mus musculus*), and fruit fly (*Drosophila melanogaster*). The transcriptional regulatory complexity is measured by using the tissue specificity of gene expression and the ratio of non-constitutively expressed to constitutively expressed genes. We demonstrate that, whereas correlation (1) holds well in the three-way comparison, correlation (2) is not true. Results from a larger dataset that includes more than 15 species, ranging from yeast to human, also reject correlation (2). The reason for the failure of correlation (2) may be ascribed to: Firstly, longer 5'UTRs do not contribute to increased tissue specificity of gene expression. Secondly, the increased numbers of common translational regulatory elements in longer 5'UTRs do not lead to increased organismal complexity.

**Conclusions:**

Our study has extended the evidence base for the correlation between organismal complexity and transcriptional regulatory complexity from mammals to fruit fly, the representative model organism of invertebrates. Furthermore, our results suggest that the elongation of 5'UTRs alone can not lead to the increase in regulatory complexity or the emergence of organismal complexity.

## Background

The evolution of organismal complexity is a fundamental issue in biological sciences. A number of hypotheses have been proposed to explain the emergence of organismal complexity, including increases in gene/protein number [1-3], gains of noncoding regulatory elements [1,2,4,5], and expansions of biological networks [2,6]. A previous study provides evidence that human (a more complex organism) has an increased proportion of genes that are narrowly expressed (indicating increased transcriptional regulatory complexity) than mouse (a less complex organism) [7]. However, the study only compares human and mouse due to data limitations. The close relationship between the two mammalian species has restricted the applicability of the study to a small evolutionary scope. For example, we are not sure whether the suggested correlation between transcriptional regulatory complexity and organismal complexity can be extended to other vertebrates (e.g. birds or fishes) or invertebrate species. Furthermore, the source of the increased regulatory complexity in complex organisms has not been fully explained, although the elongation of 5' untranslated regions (5'UTRs) has been alluded to [7]. Since 5'UTRs are associated with both transcriptional and translational *cis*-regulations [8-10], the elongation of these non-coding regions may have contributed to increased regulatory complexity [7]. A recent analysis suggested that the length of 5'UTR was unrelated to organismal complexity [11]. However, the analysis did not discuss possible reasons for the lack of correlation. Furthermore, this analysis did not take into consideration the phylogenetic relationships among the compared species (see the discussion below about independent contrast). Therefore, we are interested in reconfirming the lack of correlation between 5'UTR length and organismal complexity and examining the potential underlying molecular mechanisms. To this end, we analyzed the 5'UTR lengths of more than 15 species ranging from yeast to human. Furthermore, to examine the relationship between transcriptional regulatory complexity and 5'UTR length, we analyzed the gene expression data of human, mouse, and fruit fly, for which multiple-tissue gene expression data are available.

Notably, there have been some discussions over how organismal complexity should be measured [12]. However, most of the proposed methods cannot be applied to our study because unbiased quantification of these measurements (e.g. functional complexity [13], number of transcription factor families [14], or phenotypic complexity [15]) for all of the compared species is difficult. Therefore, we selected the number of cell types, a generally acceptable index [16], as the measurement of organismal complexity.

We also noted that closely related species might have similar genetic features, levels of organismal complexity, and 5'UTR lengths. Such similarities may lead to overweighting of some lineages and biased correlations between biological features [17]. To reduce such biases, we employed independent contrast to correct for the compared genetic characteristics [17]. Independent contrast considers the phylogenetic distances between the compared species and adjusts the weighting of the compared biological features according to the phylogenetic tree of the compared species (see Methods).

**Figure 1:**
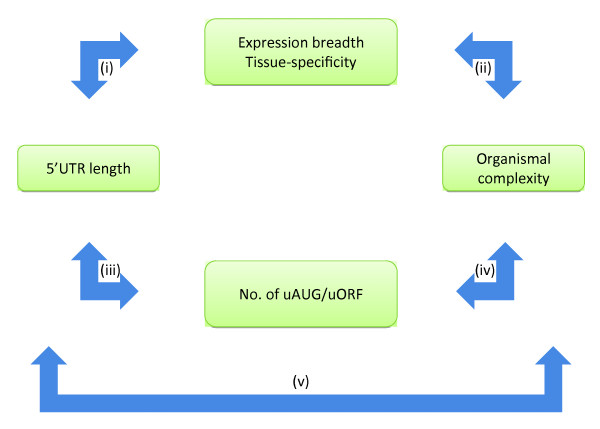
**Logical connections between the elongation of 5'UTR and the increase in organismal complexity. **The boxes are the biological characteristics that are suggested to be associated with each other, as represented by two-way arrows (i)~(v). Connection (i): longer 5'UTRs - more genes with tissue-specific expression pattern (less breadth); Connection (ii): more genes with tissue-specific expression pattern - higher organismal complexity; Connection (iii): longer 5'UTRs - more uAUGs/uORFs (upstream start codons/upstream open reading frames); Connection (iv): more uAUGs/uORFs (higher complexity in translational regulations) - higher organismal complexity; Connection (v): longer 5'UTRs - higher organismal complexity.

Figure 1 shows the conceptual framework of this study. Here we examined (A) the association between organismal complexity and the complexity in transcriptional regulation (represented by the breadth or tissue specificity of gene expression, logical connection (ii) in Figure 1), which in turn is supposedly related to the information contents (lengths) of 5'UTRs (logical connection (i)) if 5'UTR is an important contributor to organismal complexity; and (B) the relationship between organismal complexity and the abundance of 5'UTR-associated translational regulatory elements (logical connections (iii) and (iv) in Figure 1). We also examined connection (v) using several different datasets, including experimentally supported ones, to avoid potential annotation errors or dataset-specific biases. In summary, logical connection (i) posits that longer 5'UTRs contribute to higher transcriptional regulatory complexity (more non-constitutively expressed genes or more tissue-specific gene expressions); connection (ii) states that higher transcriptional regulatory complexity is related to increased organismal complexity; connection (iii) hypothesizes that longer 5'UTRs contain more translational regulatory elements (uAUGs and uORFs); connection (iv) links the increase in uAUGs and uORFs with increased organismal complexity; and connection (v) states that increased 5'UTR length contributes to increased organismal complexity.

Our results indicate that 5'UTR length correlates with neither organismal complexity nor breadth/tissue specificity of gene expression. In addition, the increased numbers of common translational regulatory signals (upstream start codons and upstream open reading frames) in longer 5'UTRs do not contribute to increased organismal complexity. In other words, we provide evidence that logical connections (i), (iv), and (v) are invalid. Therefore, we suggest that the elongation of 5'UTRs alone cannot explain the emergence of organismal complexity, despite that transcriptional regulatory complexity indeed positively correlates with organismal complexity (connection (ii)) from fruit fly to mammals.

## Results

### The increase in 5'UTR length is unrelated to the increase in organismal complexity

To examine the correlation between organismal complexity and 5'UTR length, we first selected 11 vertebrates and 3 invertebrates that have well-annotated 5'UTR information from the Ensembl website (Table 1). Yeast was also included to represent unicellular eukaryotes.

**Table 1:** The median/average 5'UTR lengths and the numbers of cell types of the compared organisms

Species	Median/Average length of 5'UTRs (bp)			**No. of cell types **^**c**^
		
	**Ensembl **^**a**^		**(C) UTRdb **^**b**^	
			
	(A)	(B)		
Human (*Homo sapiens*)	169/254	160/218	160/220	169
Chimpanzee (*Pan troglodytes*)	130/243	100/150	80/128	169
Mouse (*Mus musculus*)	126/213	120/176	131/189	159
Rat (*Rattus novegicus*)	99/168	88/130	110/180	159
Chicken (*Gallus gallus*)	75/112	77/108	80/126	154
Cow (*Bos taurus*)	92/139	89/124	95/135	159
Dog (*Canis familiaris*)	63/96	62/89	59/97	159
Frog (*Xenopus tropicalis*)	77/110	77/108	95/136	130
Zebrafish (*Danio rerio*)	104/141	106/136	109/142	120
Tetraodon (*Tetraodon nigroviridis*)	74/91	69/90	--^d^	120
Fugu (*Takifugu rubripes*)	62/96	69/102	(59/107) ^e^	120
Ascidian (*Ciona intestinalis*)	70/105	66/86	65/101	74
Fruit fly (*Drosophila melanogaster*)	127/223	125/214	131/225	64
Nematode (*Caenorhabditis elegans*)	28/68	27/54	31/70	28.5
Honeybee (*Apis mellifera*)	--	--	78/171	64
Mosquito (*Anopheles gambiae*)	--	--	125/173	64
Thale cress (*Arabidopsis thaliana*)	--	--	101/140	27.25
Rice (*Oryze sative*)	--	--	118/221	27.25
Yeast (*Saccharomyces cerevisiae*) ^f^	62/97	61/93	62/97	3.05

^a ^Two criteria were used to select a transcript for each gene with multiple isoforms: (A) a randomly selected transcript; and (B) the transcript with a pure 5'UTR.

^b ^For the UTRdb dataset, randomly selected transcripts were used in the case of alternative splicing.

^c ^The numbers of cell types were retrieved from referenced [16].

^d ^not available.

^e ^Fugu was not included in the UTRdb dataset because we could not find adequate orthologous genes in Fugu to construct the phylogenetic tree for independent contrast analysis.

^f ^The data for yeast were retrieved from Nagalakshmi et al's study [37].

**Figure 2:**
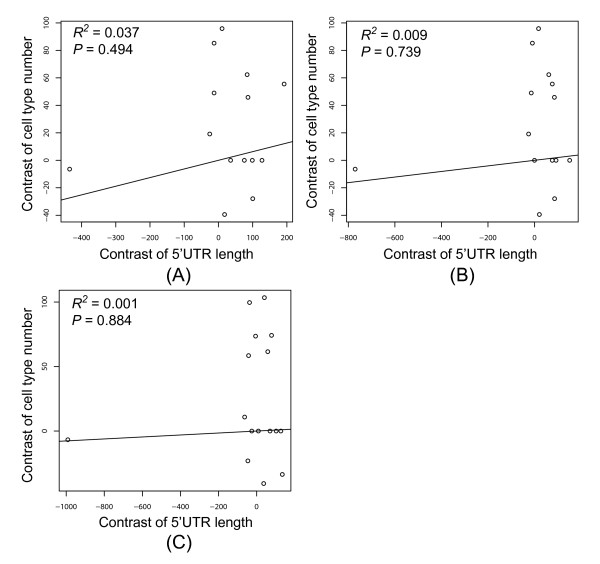
The **independent contrast-corrected correlation between the median 5'UTR length and the number of cell types based on (A) the Ensembl dataset with randomly-selected transcripts; (B) the Ensembl dataset with transcripts with pure 5'UTRs; and (C) the UTRdb dataset (only randomly selected transcripts were analyzed). **"*R*" refers to the Spearman's rank correlation coefficient. The correlation was forced to a zero-intercept linear model.

As different alternatively spliced transcripts may have different 5'UTR lengths, the selection of transcript isoforms may affect our results. Therefore, we used two different criteria to select a representative transcript in the case of alternative splicing: a randomly selected transcript or the transcript with a "pure" 5'UTR (see Methods). As shown in Figure 2, the independent contrast analyses indicate that 5'UTR length has no significant correlation with the number of cell types for either dataset (*R*^2 ^= 0.037, *P *= 0.494 for dataset (A), and *R*^2 ^= 0.009, *P *= 0.739 for dataset (B)).

Since the lengths of 5'UTRs may differ between different annotation systems, and plants are not included in the above analysis, we used an independent dataset (UTRdb, see Methods) and added two plant species to again evaluate the correlation between 5'UTR length and organismal complexity. Accordingly, the 5'UTRs of a total of 17 species, including 9 vertebrates, 5 invertebrates, 2 plants, and yeast, were analyzed (Table 1). The correlation between organismal complexity and 5'UTR length is again statistically insignificant (*R*^2 ^= 0.001, *P *= 0.884; Figure 2(C)).

To control for the factor of lineage-specific gains/losses of genes, we extracted one-to-one orthologous genes from 11 vertebrate species from the Ensembl dataset and performed the analysis again. Note that we included only vertebrate species to ensure a large enough number of genes for the analysis. The correlation remains statistically insignificant (Additional file 1), suggesting that lineage-specific gene gains/losses do not affect our result. Therefore, connection (v) in Figure 1 is not supported by this multiple-species comparison.

**Figure 3:**
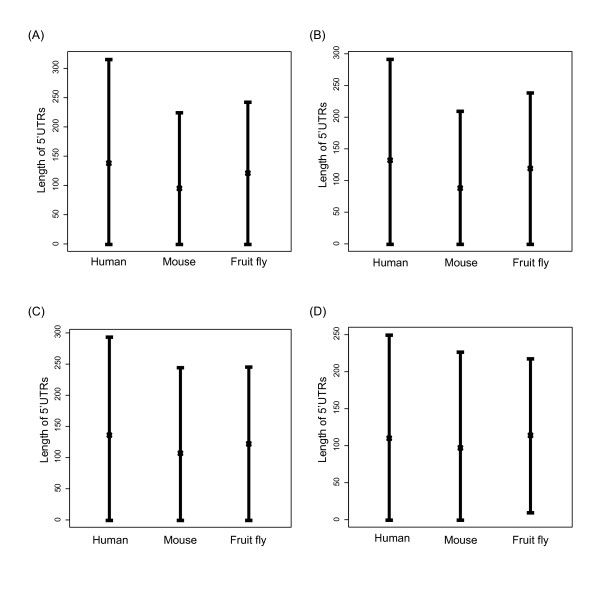
**The median 5'UTR lengths of human, mouse, and fruit fly one-to-one orthologous genes according to different data sources (the range represents twice the median absolute median)**. (A) the Ensembl dataset with randomly selected transcripts; (B) the Ensembl dataset with transcripts that have a pure 5'UTR; (C) the UTRdb dataset with randomly selected transcripts; (D) the RefSeq-CAGE dataset with randomly selected transcripts. Note that the start sites of all of the 5'UTRs in dataset (D) must be supported by the CAGE experiments [18-20]. Also note that the numbers of cell types are 169, 159, and 64, respectively, for human, mouse, and fruit fly [16].

One potential caveat in the above analyses is that the lengths of 5'UTRs may be subject to annotation errors, particularly for less extensively studied species. To tackle this problem, we compared the lengths of 5'UTRs of the one-to-one orthologous genes of three intensively studied species, namely human, mouse, and fruit fly with reference to three different databases: Ensembl, UTRdb, and RefSeq-CAGE (RefSeq transcripts with the 5'cap annotation supported by CAGE data [18-20]) (Figure 3; Methods). For the UTRdb and RefSeq-CAGE datasets, a randomly selected transcript for each gene was analyzed, whereas for the Ensembl dataset, randomly selected and pure-5'UTR transcripts were separately analyzed. As is commonly recognized, the numbers of cell types (Table 1) indicate that human is the most complex organism among the three, followed by mouse, and finally by fruit fly. The 5'UTR lengths of the three organisms are expected to follow the same order if organismal complexity is indeed associated with 5'UTR length. Unexpectedly, however, fruit fly actually has longer 5'UTRs than mouse in all of the analyzed datasets (All *P *values < 1.3E-20 by the Mann-Whitney *U *test; Figure 3). In the RefSeq-CAGE dataset, fruit fly has the longest 5'UTRs (*P *< 4.9E-9 in both human-fly and mouse-fly comparisons; Figure 3(D)) despite its lowest organismal complexity. Meanwhile, in the other three datasets, the differences in 5'UTR length between human and fruit fly are all statistically insignificant (Figure 3 (A)~3(C)). These observations suggest that the increase in organismal complexity is not directly related to the elongation of 5'UTR. Therefore, connection (v) in Figure 1 is again not supported by the high-quality data.

### The length of 5'UTR cannot fully explain the breadth or tissue specificity of gene expression

We have shown that organismal complexity does not increase with increasing length of 5'UTR. We then examine possible reasons for the lack of correlation by investigating logical connections (i), (ii), (iii), and (iv) in Figure 1 using the biological features of the three intensively studied species, for multiple-tissue (>10 tissues) gene expression data are available only for these species. We first analyzed the relationship between 5'UTR length and the breadth/tissue specificity of gene expression. Vinogradov and Anatskaya [7] showed that human had a higher fraction of non-constitutively expressed genes than mouse, which was suggested to result from human's longer 5'UTRs (logical connections (i) and (ii) in Figure 1). In this vein, organisms with longer 5'UTRs are expected to have a larger proportion of narrowly expressed genes (higher tissue specificity) because the supposedly larger numbers of regulatory elements in longer 5'UTRs allow subtle transcriptional regulations, which should in turn lead to increased organismal complexity.

To examine the validity of these logical connections, we compared the expression patterns of one-to-one orthologous gene among the three species for all the available tissues (Methods [21,22]). Notably, there are two technical issues in this comparison. First, the numbers of experimentally examined tissues are much larger for mammals (79 for human and 61 for mouse) than for fruit fly (17 tissues). This may lead to a larger proportion of "constitutively expressed genes" in fruit fly than in mammals because, intuitively, a gene is more likely to be expressed in 17 tissues than in 61 (or 79) tissues. Second, it is infeasible to compare "homologous" tissues between mammals and fruit fly. To address these issues, we randomly sampled 10 non-redundant tissues from each of the species 1,000 times, and analyzed the expression profiles in the sampled tissues (Methods). The rationale of this analysis is that the gene expression patterns in complex organisms should be more variable than in relatively simple organisms. In other words, given the same numbers of tissues, more complex organisms should have fewer genes that are expressed in all of the examined tissues, and demonstrate higher levels of tissue specificity of gene expression. We then took three measurements for the analyzed genes in the sampled tissues: (a) the 5'UTR length; (b) the ratio of "non-constitutively expressed genes" to "constitutively expressed" genes (Methods); and (c) the mean of tissue specificity of gene expression (the "τ" statistic [23]).

**Figure 4:**
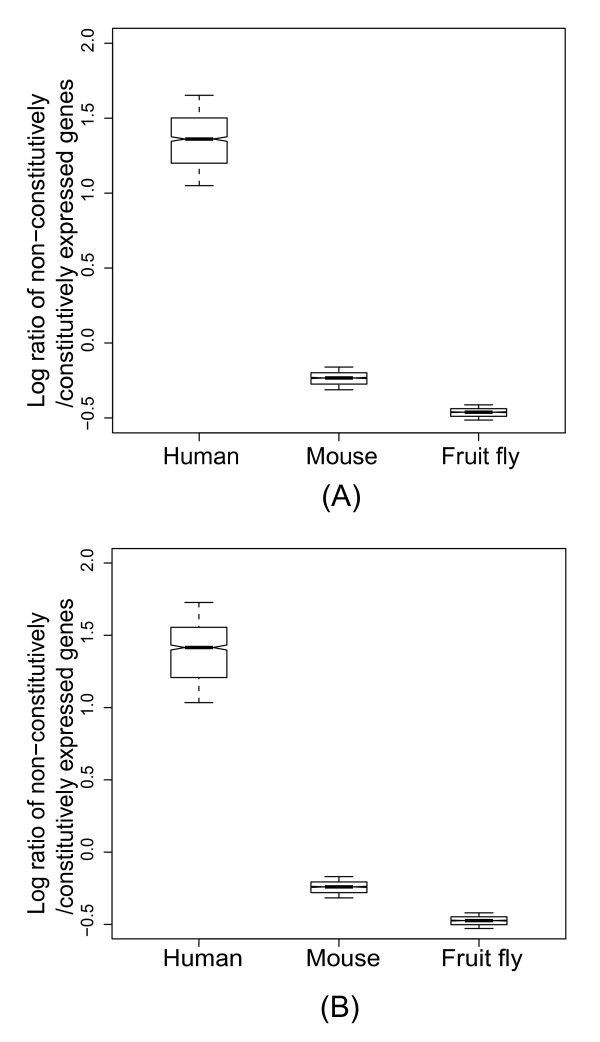
**Distribution of the ratios (log 2 scale) of non-constitutively to constitutively expressed genes. **(A) the Ensembl dataset with randomly-selected transcripts; (B) the Ensembl dataset with transcripts with pure 5'UTRs. The distributions were derived from 1,000 re-samplings to obtain 10 non-redundant tissues for each species. All of the pairwise differences are statistically significant (*P *≦ 1.94E-192, Mann-Whitney *U *test).

**Figure 5:**
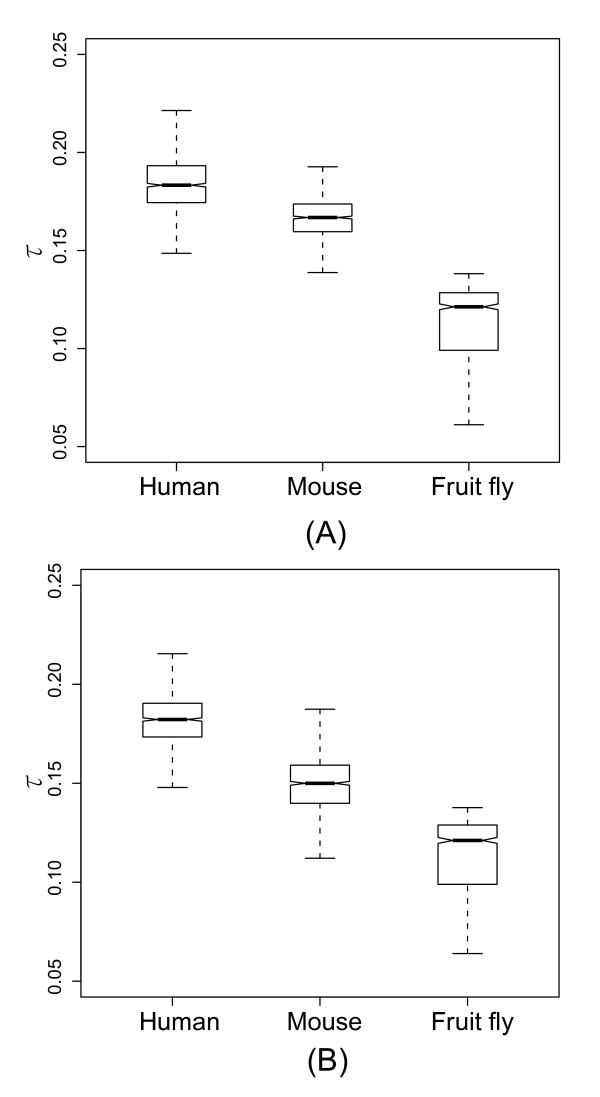
**Distribution of the average τ values. **(A) the Ensembl dataset with randomly-selected transcripts; (B) the Ensembl dataset with transcripts with pure 5'UTRs. The distributions were derived from 1,000 re-samplings to obtain 10 non-redundant tissues for each species. All of the pairwise differences are statistically significant (*P *≦ 2.54E-201, Mann-Whitney *U *test).

With the above data, we were able to examine and potentially extend from mammals to fruit fly the notion that more complex organisms have a larger proportion of narrowly expressed genes [7]. According to this hypothesis, human is expected to have the largest fraction of non-constitutively expressed genes, followed by mouse, and lastly by fruit fly, which is in fact supported by our result (Figure 4). The tissue specificity of gene expression of the three species also shows a similar trend (Figure 5). Note that we also re-sampled 17 tissues from the human and mouse data 1,000 times and compare the distributions with the median values of the fruit fly data. The results remain the same (Additional files 2 and 3). Therefore, our results indicate that logical connection (ii) in Figure 1 is applicable to such a wide range as from fruit fly to mammals.

**Figure 6:**
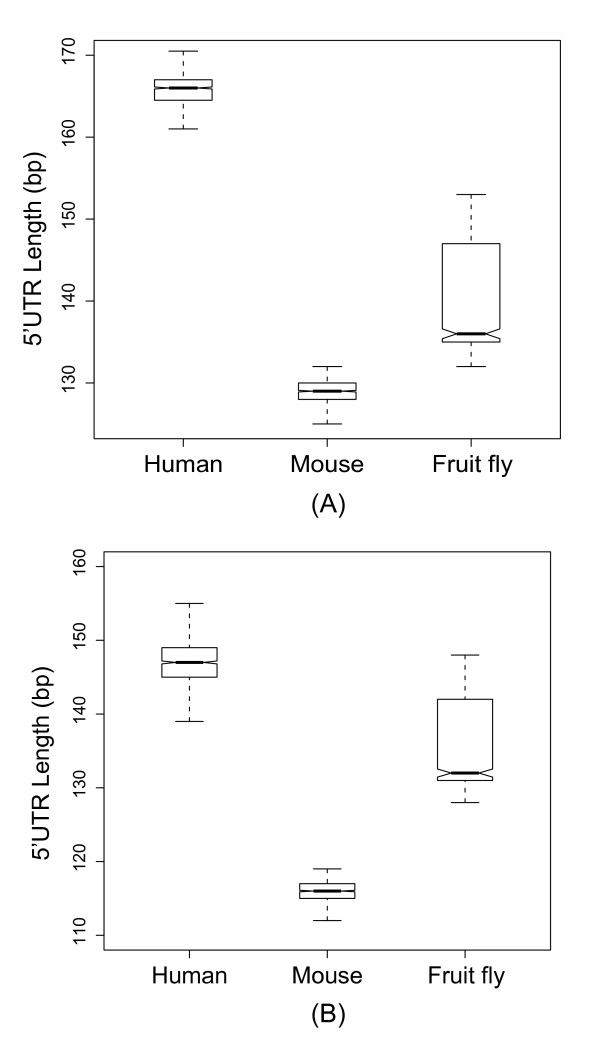
**Distribution of the median 5'UTR lengths. **(A) the Ensembl dataset with randomly-selected transcripts; (B) the Ensembl dataset with transcripts with pure 5'UTRs. The distributions were derived from the genes that were expressed in the randomly sampled tissues in Figures 2 and 3. All of the pairwise differences are statistically significant (*P *≦ 8.47E-276, Mann-Whitney *U *test)

We then examined logical connection (i). If the elongation of 5'UTR actually contributes to the increase in transcriptional regulatory complexity, it is expected that human has the longest 5'UTRs and fruit fly has the shortest for the genes that are expressed (both constitutively and non-constitutively expressed) in the re-sampled tissues (see Methods for the definition of expressed genes). This inference is based on the observation that these "expressed genes" exhibit highest tissue specificity in human and lowest specificity in fruit fly (Figures 4 and 5). However, this expected result is not observed. Among the examined genes, fruit fly in fact has significantly longer 5'UTRs than mouse (Figure 6). A potential caveat in this analysis is that the fruit fly and mammalian expression data were generated by two different groups at different times. In addition, the cutoff thresholds of "expressed genes" are different between mammals and fruit fly (Methods). The longer 5'UTRs in fly than in mouse thus may result from data bias. However, a similar trend is actually observed in the all-ortholog comparison (Figure 3). Therefore, our results may have reflected the biological truth, arguing against logical connection (i). In other words, organisms with longer 5'UTRs do not necessarily have more non-constitutively expressed genes.

### Increasing numbers of upstream start codons and upstream open reading frames do not contribute to increase in organismal complexity

Next, we examine the relationship between organismal complexity and the numbers of translational regulatory motifs in 5'UTRs (logical connections (iii) and (iv) in Figure 1). Here we use two common motifs, namely upstream start codons (uAUGs) and upstream open reading frames (uORFs), to represent the translational regulatory elements in 5'UTRs. This is reasonable because these elements occur frequently in 5'UTRs and can significantly down-regulate the translation of the main coding regions [24]. Furthermore, for the same species, the numbers of uAUGs and uORFs are positively correlated with the lengths of 5'UTRs [25,26]. We can examine whether this is also true between different species. To this end, we used the 15-species Ensembl datasets to examine the correlation between 5'UTR length and the number of uAUG/uORF. In fact, the numbers of uAUGs and uORFs are both positively correlated with the lengths of 5'UTRs, with only one exception (the number of uAUGs VS. 5'UTR length for randomly selected transcripts; Additional file 4). Therefore, the general trend is that organisms with longer 5'UTRs tend to have more translational regulatory elements, which supports logical connection (iii) in Figure 1.

We also use the Ensembl datasets to examine whether the numbers of uAUG/uORF correlate with organismal complexity. The independent contrast analyses indicate that the number of neither of the two types of regulatory elements per gene significantly correlates with organismal complexity (*P *≧ 0.340; Additional file 5). Therefore, logic connection (iv) is not supported.

In sum, we provide evidence against two important assumptions (connections (i) and (iv) in Figure 1) in the 5'UTR length-organismal complexity hypothesis. The failure of these assumptions leads to falsification of the hypothesis itself (connection (v)). Therefore, we suggest that the elongation of 5'UTR is not the major contributor of the increased organismal complexity.

## Discussion

We have demonstrated that the elongation of 5'UTR is not directly related to the increase in organismal complexity among human, mouse, and fruit fly (and also in several larger datasets). The possible reason for the lack of correlation is twofold. First, at the transcription level, 5'UTR length is not correlated with breadth/tissue specificity of gene expression. Second, at the translation level, the larger numbers of common translational regulatory elements in longer 5'UTRs do not lead to increased organismal complexity.

However, we emphasize that our results support the correlation between organismal complexity and the complexity in gene regulations [7]. It is well established that transcriptional/translational regulations involve a wide variety of *trans*- and *cis*- factors. 5'UTRs represent only part of the *cis*-factors. We cannot rule out the possibility that organismal complexity is associated with the interactions between 5'UTRs and other regulatory factors, thus blurring the correlation between 5'UTR length and organismal complexity. Furthermore, 5'UTRs may contain so far uncharacterized transcriptional/translational regulatory elements, which alone or in combination with other regulatory elements may contribute to organismal complexity.

The apparent lack of correlation between 5'UTR length and organismal complexity is unexpected, for the elongation of 5'UTRs and the emergence of organismal complexity were suggested to result from the same evolutionary process [27,28]. It has been proposed that the decrease in population size and the consequent reduction of selective constraint on genome evolution led to the accumulation of regulatory elements and the emergence of organismal complexity [29]. Therefore, it appears reasonable to assume an association between organismal complexity and 5'UTR length. However, as we discussed earlier, 5'UTR is not the only regulatory element in the genome. For example, non-coding RNA-mediated gene regulations [30-32], nonsense-mediated decay [33], the lengths and interactions of protein coding sequences [34], and 3'UTRs may all contribute to regulatory complexity [11]. To be sure, 5'UTRs represent only part of the complicated machinery of eukaryotic gene regulations. The proportion that 5'UTRs contribute to the variations in transcriptional/translational regulations remains unknown. And such proportions are also likely to vary with biological conditions. It is intriguing to study whether the collective length of all regulatory elements correlates significantly with organismal complexity. A potential approach is to integrate these features into a multiple regression model and analyze the contributions of each characteristic to the variations in organismal complexity.

## Conclusions

Our study has extended the evidence base for the association between organismal complexity and transcriptional regulatory complexity from mammals to fruit fly. We also show that increased organismal complexity does not result directly from the elongation of 5'UTRs because longer 5'UTRs do not contribute to higher regulatory complexity. Therefore, despite the proposed common evolutionary origin of these two biological phenomena, one single type of regulatory sequence (5'UTR) may not account for such a multi-faceted feature as organismal complexity.

## Methods

### Data sources

We used two primary data sources for well-annotated 5'UTR information: Ensemble (version 56) and UTRdb (http://utrdb.ba.itb.cnr.it/; updated in July 2010) [35]. For the Ensembl dataset, 11 vertebrate and 3 invertebrate species were selected (Table 1). The sequences of 5'UTRs and gene annotations (Ensembl version 56) were retrieved by using BioMart [36]. For the UTRdb dataset, 10 vertebrate, 4 invertebrate, and 2 plant species were selected (Table 1). The 5'UTR sequences of yeast (*Saccharomyces cerevisiae*) were retrieved from a recent publication [37] and added to both datasets for subsequent analyses. Note that the Ensembl dataset was applied in all of the analyses of this study, whereas the UTRdb dataset was used to examine logical connection (v) only.

Furthermore, we selected the most extensively studied species, namely human (*Homo sapiens*), mouse (*Mus musculus*), and fruit fly (*Drosophila melanogaster*) for analyses. The high study intensities for these species have considerably reduced the probability of annotation errors as compared with the other analyzed species. In addition, the large-scale gene expression data available for these three species enable us to analyze the correlation between 5'UTR length and gene expression patterns, which would be impossible for the other species. In addition to Ensemble and UTRdb data, a RefSeq-CAGE dataset was also employed. For this last dataset, only the RefSeq-annotated transcription start sites that were supported by the CAGE tag clusters [18-20] were retained. Therefore, the lengths of 5'UTRs derived from this dataset were considered as highly accurate. Chromosomal positions of tag clusters were downloaded from the FANTOM website for human and mouse [18,19] and from a recent genome-wide study for fruit fly [20].

To further enhance the quality of the data, several criteria were applied to filter the retrieved transcripts: the transcripts to be analyzed must (a) have an annotated 5'UTR; (b) be a known transcript (rather than a novel or predicted transcript); and (c) have a known protein product. The last two conditions were employed to ensure that the 5' and 3' termini of the analyzed 5'UTRs were experimentally supported. In the case of alternative splicing, we used two different criteria to select one transcript for each gene for the Ensembl dataset (Table 1): (A) a randomly selected transcript; or (B) the transcript with a "pure 5'UTR" (i.e. a 5'UTR that does not overlap with the coding sequences in any other transcripts). In the latter case, we further filtered out the pure 5'UTRs that matched any of the entries in the non-redundant (NR) protein database (ftp://ftp.ncbi.nih.gov/blast/db/FASTA/nr.gz) by using blastx [38] with the default parameters (*E*-value < 10^-5^). Analyses of both datasets yield consistent results. For the UTRdb dataset, we randomly selected one transcript for each gene with alternatively spliced isoforms.

For the measurement of organismal complexity, we used the number of cell types because this indicator has been shown to be highly correlated with organismal complexity [39]. The numbers of cell types of the compared species (Table 1) were retrieved from Vogal and Chothia's study [16].

### Evaluating the correlation between organismal complexity and genetic features

The genetic characteristics of closely related species may not evolve independently, which may lead to biased correlations between genetic features [17]. To eliminate such biases, we employed the "CONTRAST" module of PHYLIP [17] to derive the contrasts of the measured biological features (5'UTR lengths, the numbers of translational regulatory elements, and the numbers of cell types) with reference to the phylogenetic tree of the compared organisms. The process is summarized as follows. First, the phylogenetic tree was constructed based on the protein sequences of one-to-one orthologous genes of the compared species. Second, unweighted contrasts of the biological characteristics (e.g. 5'UTR length) were calculated for the internal nodes of the phylogenetic tree. Third, weighted contrasts were calculated according to the genetic distances between the nodes of the tree.

The Spearman's correlations of a zero-intercept linear regression model were then evaluated for the derived contrasts of biological characteristics by using the R program (http://www.r-project.org). The reason for using the zero-intercept regression is that no changes in one biological characteristic are expected if the other characteristic does not change (e.g. no changes in organismal complexity are expected if the lengths of 5'UTRs do not change). Notably, the overall results hold well even if we use the regular Spearman's correlation.

### Measurements of gene expression breadth and tissue specificity

Gene expression data of human and mouse were retrieved from the BioGPS website (http://biogps.gnf.org/downloads/). The datasets covered 79 human and 61 mouse tissues, where the levels of gene expression were measured using the Affymetrix microarray chips (U133A/GNF1H for human and GNF1M for mouse) [21]. To determine the probe-gene associations, we blastn-aligned [38] the probe sequences against the complementary DNA (cDNA) sequences of known human and mouse protein coding genes retrieved from Ensembl version 56. Only the probes that could be completely matched to a cDNA with 100% identity were retained. The probes that matched more than one gene were excluded. In the cases where multiple probes matched the same gene, we retained the probe that had the highest sum of expression levels in all tissues. Accordingly, 15,834 human and 15,627 mouse genes were identified and subsequently analyzed. The gene expression data of adult fruit fly were retrieved from the FlyAtlas (http://flyatlas.org/drosophila_2.na23.annot.csv), which covered 17 tissues that were examined using the Affymetrix Drosophila Genome 2.0 Array [22]. The probe-gene associations were determined as described above. Accordingly, 12,095 of the fruit fly genes were included in the subsequent analyses.

Note that the numbers of examined tissues differ remarkably between the mammalian species and fruit fly. To fairly reflect the differences in expression patterns among human, mouse, and fruit fly, we randomly sampled 10 non-redundant tissues from each of species (or 17 tissues from human and mouse) 1,000 times, and analyzed the expression profiles in the sampled tissues. A mammalian gene was considered as expressed in a given tissue if its average difference (AD) value was larger than 200 [21]. In the case of fruit fly, a gene was regarded as expressed if it had at least 3 present calls out of 4 biological replicates [40]. The genes that were not expressed in any of the 10 (or 17) sampled tissues were excluded. We then took three measurements for the analyzed genes in the sampled tissues: (a) the median 5'UTR lengths; (b) the ratio of "non-constitutively expressed genes" (defined as genes that were not expressed in all of the 10 (or 17) sampled tissues) to "constitutively expressed" genes (genes that were expressed in all of the 10 or 17 sampled tissues); and (c) the average tissue specificity of gene expression. Tissue specificity of gene expression was measured by the modified τ statistic [23], which considered both expression breadth and expression level of a gene. The τ value falls between 0 and 1. A larger τ value indicates higher tissue specificity of gene expression.

### Identification of translational regulatory elements in 5'UTRs

Identification of all of the translational regulatory elements in 5'UTRs is infeasible due to our limited understanding of these elements. Instead, we calculated the numbers of two common regulatory elements that have been proved able to significantly alter the levels of protein translation: upstream start codons (uAUGs) [41] and upstream open reading frames (uORFs) [24]. The uAUGs in 5'UTRs were scanned from the 5' cap to the 3' end in three different reading frames. A uORF was defined as a putative open reading frame that started at a uAUG and terminated at a stop codon within a 5'UTR. A uORF must be at least 9 nucleotides long, including a uAUG, a stop codon, and at least one codon in-between. To avoid redundancy in the calculation of uORF numbers, only the first uAUG triplet was used as the start of a uORF when multiple in-frame uAUGs were present.

This study applies only bioinformatics analyses on data from the public domain. Therefore, no ethical approval or consent for data usage is required.

## List of abbreviations

5'UTR: 5' untranslated region; CAGE: cap analysis of gene expression; uAUG: upstream start codon; uORF: upstream open reading frame.

## Competing interests

The authors declare that they have no competing interests.

## Authors' contributions

HYL analyzed 5'UTR length, and the numbers of uAUGs/uORFs. CLP carried out the analysis of expression data. HYL and CLP performed all the statistical tests. CHC participated in the design of the study, data analyses, and drafted the manuscript. FCC conceived the study, and participated in its design and coordination and drafted the manuscript. All authors have read and approved the final manuscript.

## Supplementary Material

Additional file 1 **The correlation between 5'UTR length and the number of cell types according to the one-to-one orthologous genes of eleven vertebrate species**. The correlation was corrected using independent contrast. The eleven vertebrate species are listed in Table 1 (Human~Fugu). Click here for file

Additional file 2 **Distribution of the ratios (log 2 scale) of non-constitutively to constitutively expressed genes **(A) the Ensembl dataset with randomly-selected transcripts; (B) the Ensembl dataset with transcripts with pure 5'UTRs. The distributions were derived from 1,000 re-samplings to obtain 17 non-redundant tissues for human and mouse. Since fruit fly has only 17 tissues, no re-sampling was performed. The dashed line indicates the median value for the fruit fly dataset. Click here for file

Additional file 3 **Distribution of the average τ values **(A) the Ensembl dataset with randomly-selected transcripts; (B) the Ensembl dataset with transcripts with pure 5'UTRs. The distributions were derived from 1,000 re-samplings to obtain 17 non-redundant tissues for human and mouse. Since fruit fly has only 17 tissues, no re-sampling was performed. The dashed line indicates the median value for the fruit fly dataset. Click here for file

Additional file 4 **The independent contrast-corrected correlation between 5'UTR length and the number of uAUGs/uORFs**. (A) and (C) show the independent contrast-corrected correlation between the number of uAUGs and 5'UTR length; (B) and (D) show the correlation between the number of uORFs and 5'UTR length. Note that the left panel ((A) and (C)) is based on the Ensemble dataset with randomly selected transcripts, while the right panel ((B) and (D)) is based on the Ensemble dataset with transcripts with pure 5'UTRs. Click here for file

Additional file 5 **The independent contrast-corrected correlation between the number of cell types and the number of uAUGs/uORFs**. (A) and (C) show the independent contrast-corrected correlation between the number of uAUGs and the number of cell types; (B) and (D) show the correlation between the number of uORFs and the number of cell types. Note that the left panel ((A) and (C)) is based on the Ensemble dataset with randomly selected transcripts, while the right panel ((B) and (D)) is based on the Ensemble dataset with transcripts with pure 5'UTRs. Click here for file
