# Supplementary material for: The plausible reason why the length of 5' untranslated region is unrelated to organismal complexity
Source: BMC Res Notes. 2011 Aug 27;4:312. doi: 10.1186/1756-0500-4-312 (PMC3224463; doi:10.1186/1756-0500-4-312)
Supplement: Additional file 1 — The correlation between 5'UTR length and the number of cell types according to the one-to-one orthologous genes of eleven vertebrate species. The correlation was corrected using independent contrast. The eleven vertebrate species are listed in Table 1 (Human~Fugu). [file 1756-0500-4-312-S1.PDF]

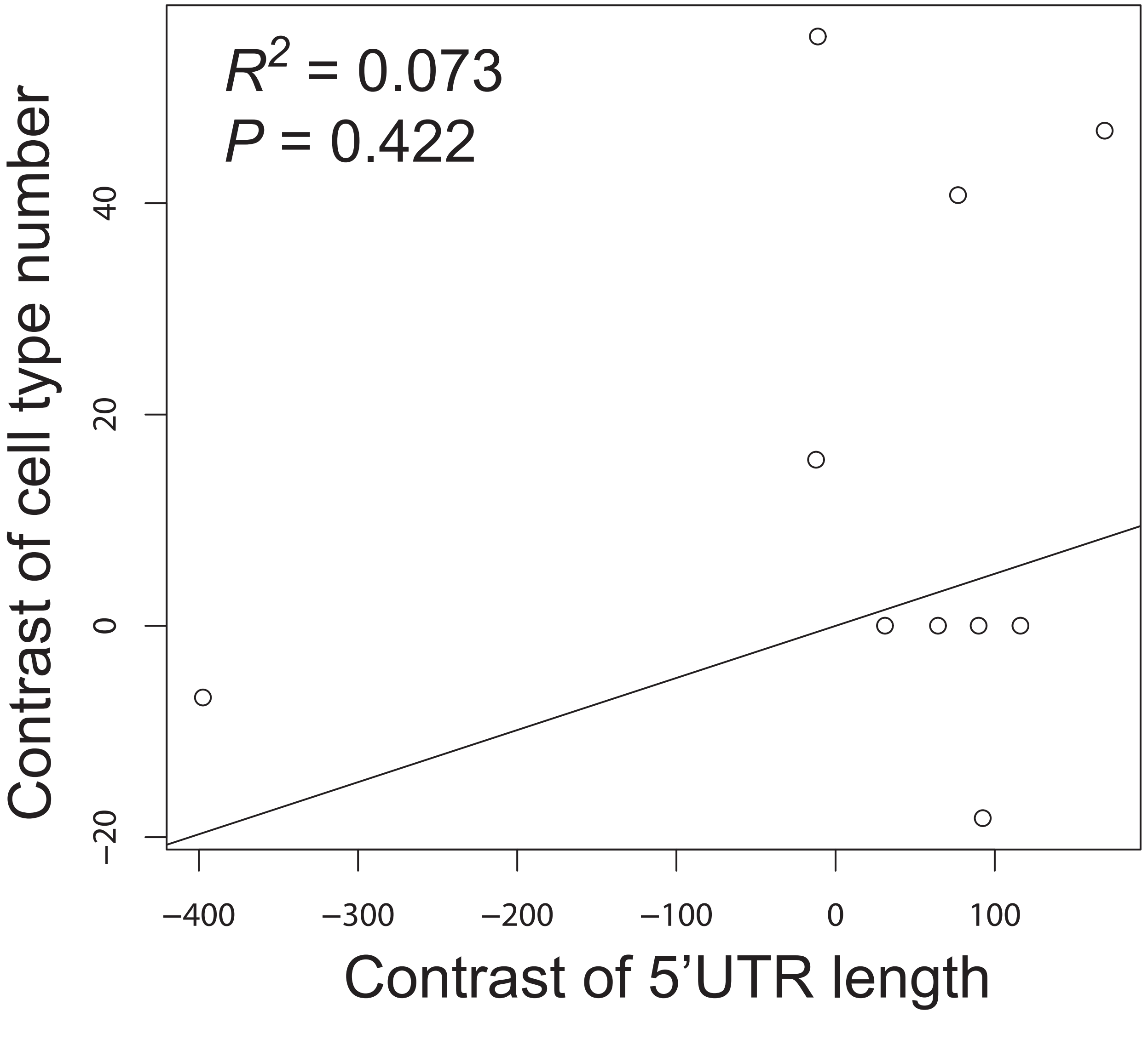

Additional file 1. The indepedent contrast-corrected correlation between 5’UTR length and the number of cell types based on one-to-one orthologous genes retrieved from 11 vertebrates species from the Ensembl dataset.
