# Supplementary material for: The plausible reason why the length of 5' untranslated region is unrelated to organismal complexity
Source: BMC Res Notes. 2011 Aug 27;4:312. doi: 10.1186/1756-0500-4-312 (PMC3224463; doi:10.1186/1756-0500-4-312)
Supplement: Additional file 2 — Distribution of the ratios (log 2 scale) of non-constitutively to constitutively expressed genes (A) the Ensembl dataset with randomly-selected transcripts; (B) the Ensembl dataset with transcripts with pure 5'UTRs. The distributions were derived from 1,000 re-samplings to obtain 17 non-redundant tissues for human and mouse. Since fruit fly has only 17 tissues, no re-sampling was performed. The dashed line indicates the median value for the fruit fly dataset. [file 1756-0500-4-312-S2.PDF]

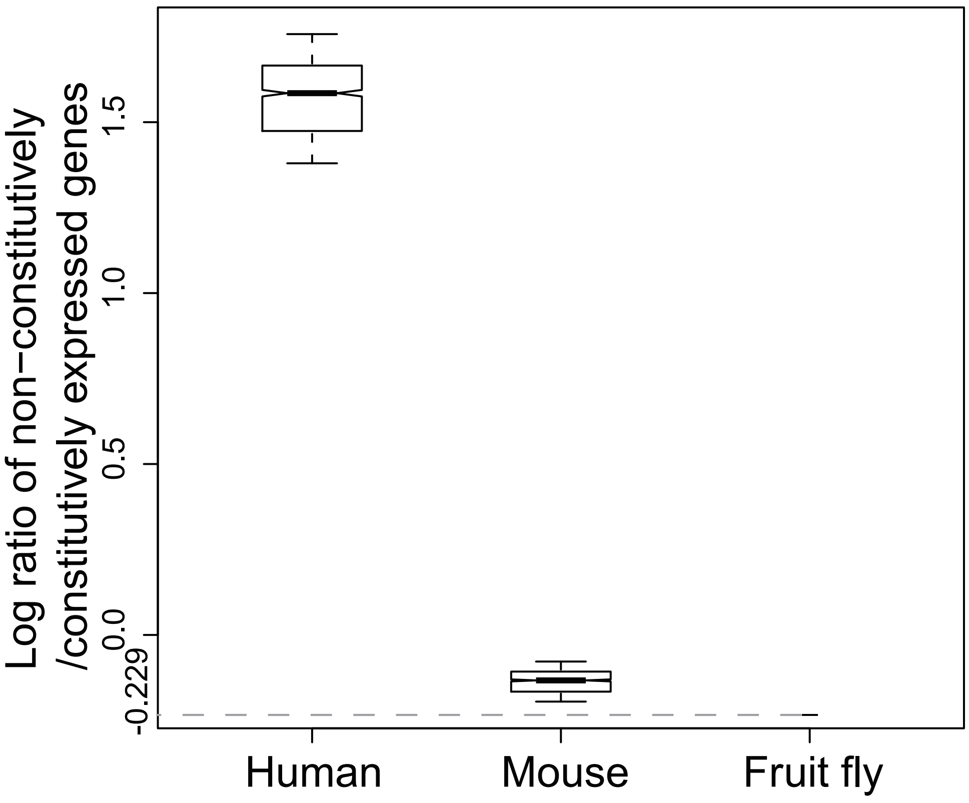

(A)

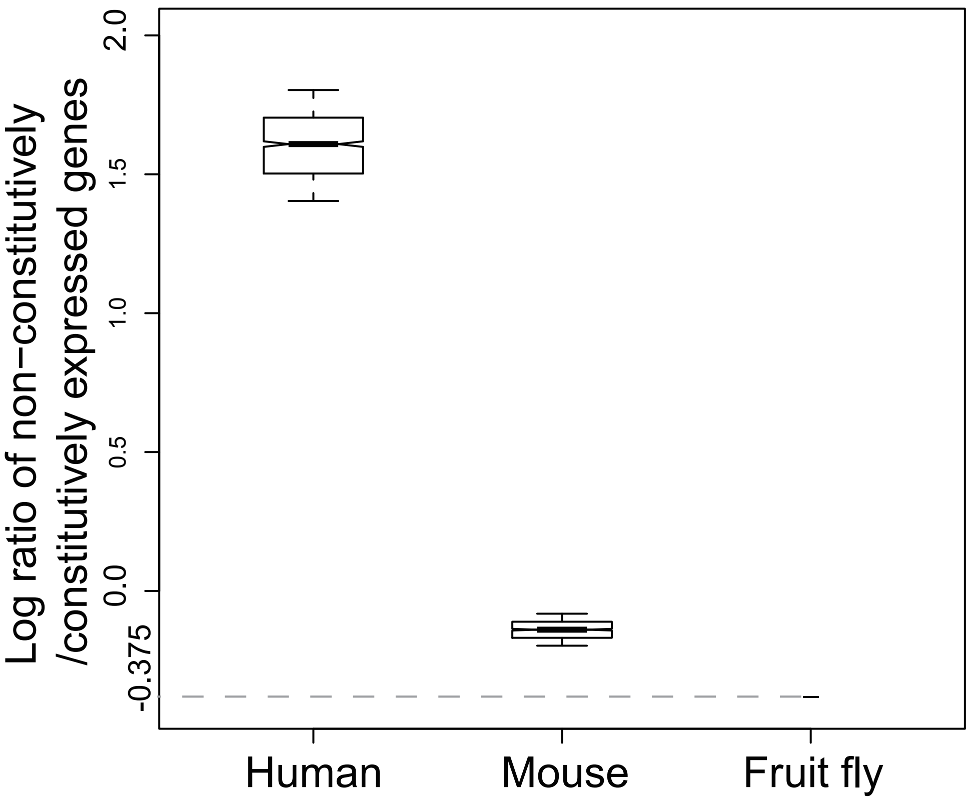

(B)

Additional file 2. Distributions of the ratios (log2 scale) of non-constitutively to consitutively expressed genes. (A) the Ensembl dataset with randomly-selected transcripts; (B) the Ensembl dataset with transcripts with pure 5'UTRs. The distributions were derived from 1,000 re-samplings to obtain 17 non-redundant tissues for human and mouse. Since fruit fly has only 17 tissues, no re-sampling was performed. The dashed line indicates the median value for the fruit fly dataset.
