# Supplementary material for: The plausible reason why the length of 5' untranslated region is unrelated to organismal complexity
Source: BMC Res Notes. 2011 Aug 27;4:312. doi: 10.1186/1756-0500-4-312 (PMC3224463; doi:10.1186/1756-0500-4-312)
Supplement: Additional file 4 — The independent contrast-corrected correlation between 5'UTR length and the number of uAUGs/uORFs. (A) and (C) show the independent contrast-corrected correlation between the number of uAUGs and 5'UTR length; (B) and (D) show the correlation between the number of uORFs and 5'UTR length. Note that the left panel ((A) and (C)) is based on the Ensemble dataset with randomly selected transcripts, while the right panel ((B) and (D)) is based on the Ensemble dataset with transcripts with pure 5'UTRs. [file 1756-0500-4-312-S4.PDF]

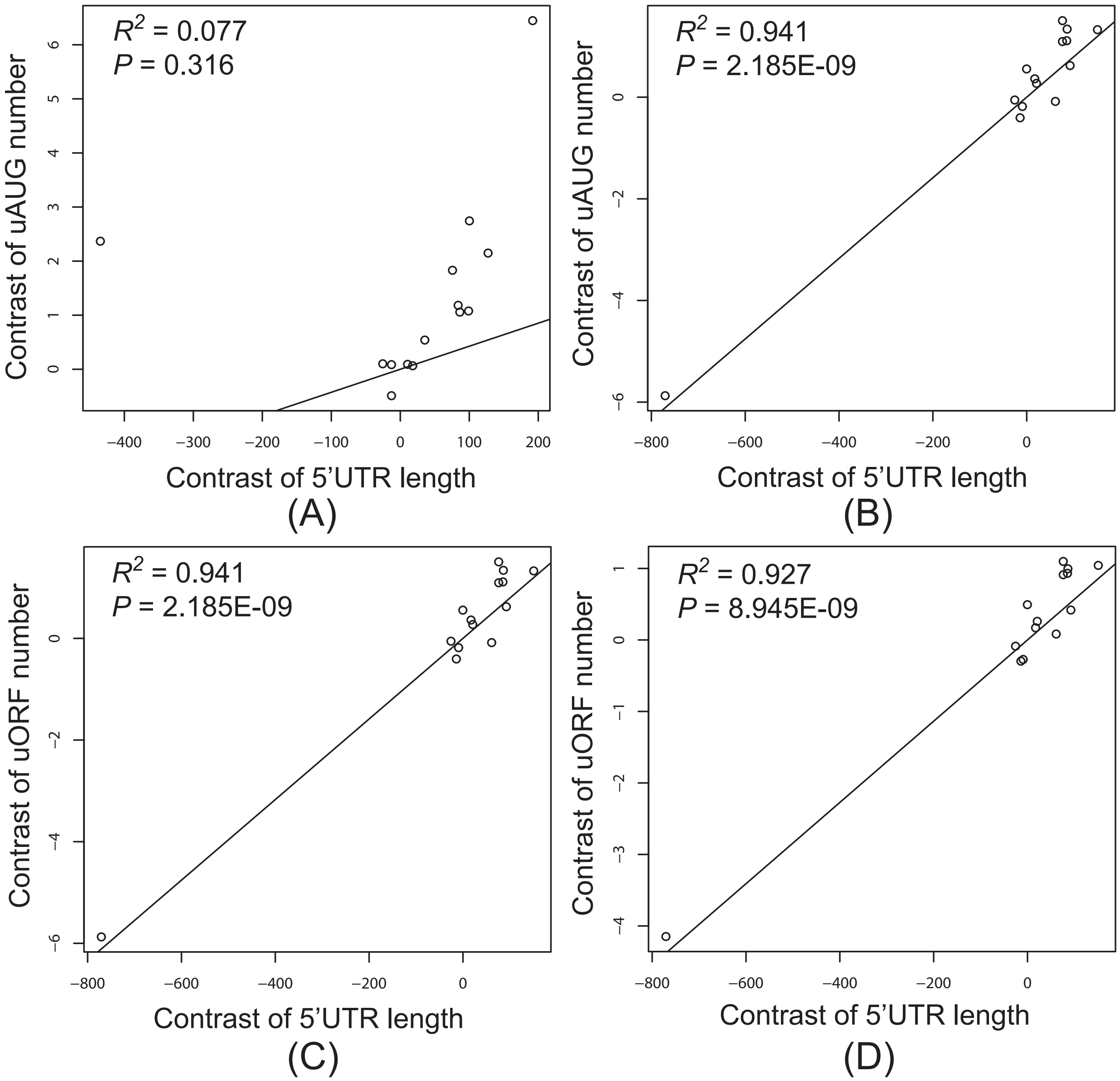

Additional file 4. The indepedent contrast-corrected correlation between the length of 5'UTR and the number of uAUGs ((A) and (B)) or th number of uORFs ((C) and (D)). Note that (A) and (C) were based on the Ensemble dataset with randomly select transcripts, whereas (B) and (D) were based on the Ensembl dataset with pure-5'UTR transcripts.
