# Supplementary material for: The plausible reason why the length of 5' untranslated region is unrelated to organismal complexity
Source: BMC Res Notes. 2011 Aug 27;4:312. doi: 10.1186/1756-0500-4-312 (PMC3224463; doi:10.1186/1756-0500-4-312)
Supplement: Additional file 5 — The independent contrast-corrected correlation between the number of cell types and the number of uAUGs/uORFs. (A) and (C) show the independent contrast-corrected correlation between the number of uAUGs and the number of cell types; (B) and (D) show the correlation between the number of uORFs and the number of cell types. Note that the left panel ((A) and (C)) is based on the Ensemble dataset with randomly selected transcripts, while the right panel ((B) and (D)) is based on the Ensemble dataset with transcripts with pure 5'UTRs. [file 1756-0500-4-312-S5.PDF]

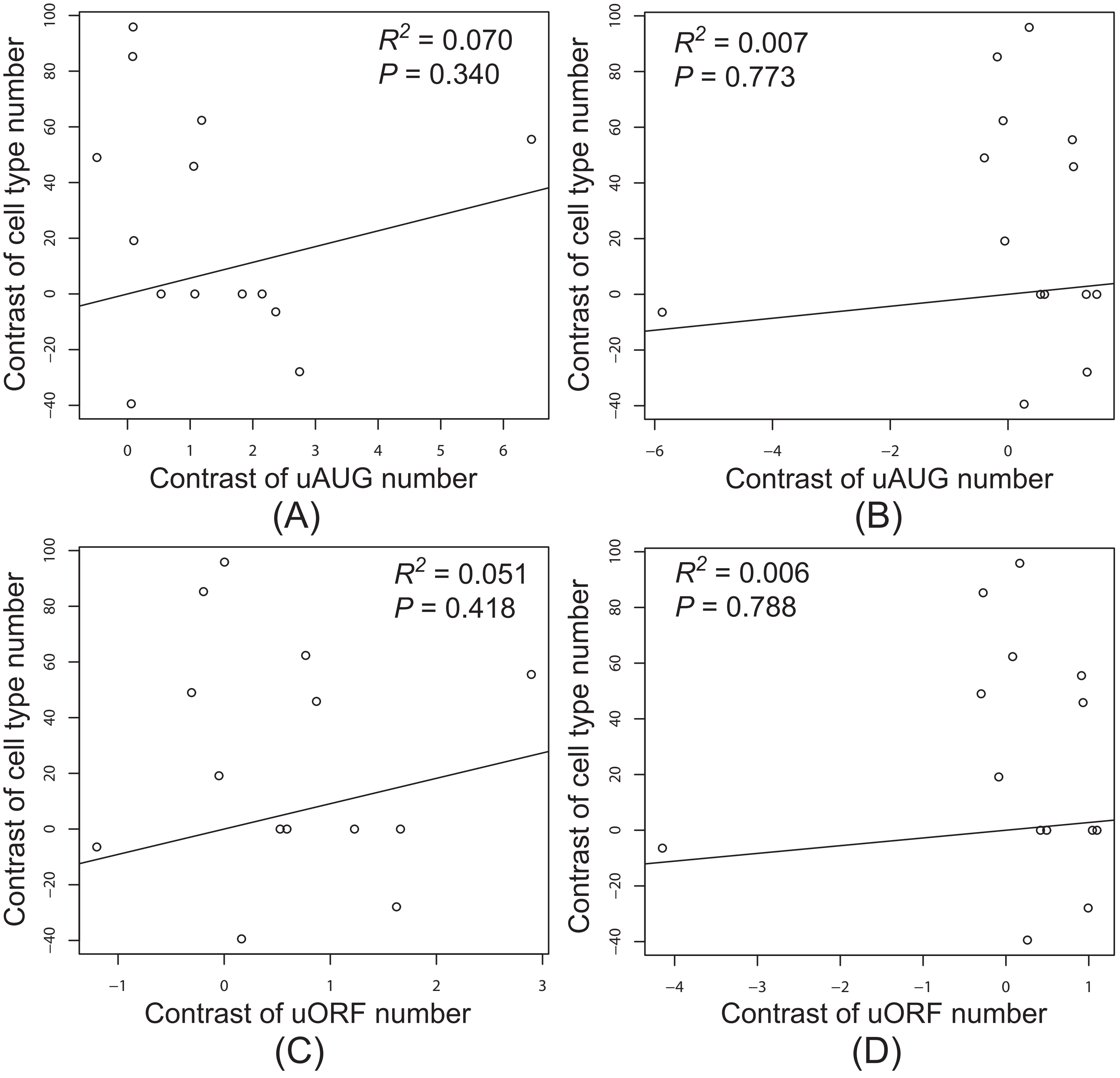


Additional file 5. The indepedent contrast-corrected correlation between the number of cell types and the number of uAUGs ((A) and (B)) or the number of uORFs per gene ((C) and (D)). Note that (A) and (C) were based on the Ensembl dataset with randomly selected transcripts, whereas (B) and (D) were based on the Ensemble dataset with pure 5’UTRs.
